# Perioperative Goal-Directed Therapy during Kidney Transplantation: An Impact Evaluation on the Major Postoperative Complications

**DOI:** 10.3390/jcm8010080

**Published:** 2019-01-11

**Authors:** Marco Cavaleri, Massimiliano Veroux, Filippo Palermo, Francesco Vasile, Mirko Mineri, Joseph Palumbo, Lorenzo Salemi, Marinella Astuto, Paolo Murabito

**Affiliations:** 1 Department of Anaesthesia and Intensive Care, “Sant’ Elia” Hospital, via L.Russo 6, 93100 Caltanissetta, Italy; 2 Vascular Surgery and Organ Transplant Unit, Department of Medical and Surgical Sciences and Advanced technologies “G F Ingrassia”, University Hospital “G.Rodolico”, University of Catania, via Santa Sofia 78, 95123 Catania, Italy; veroux@unict.it; 3 Department of Clinical and Molecular Biomedicine, University of Catania, via Palermo 636, 95123 Catania, Italy; fpalermo@unict.it; 4 School of Anaesthesia and Intensive Care, University Hospital “G.Rodolico”, University of Catania, via Santa Sofia 78, 95123 Catania, Italy; frankie.vas@hotmail.it (F.V.); mirkomineri@gmail.com (M.M.); josephpal@hotmail.it (J.P.); salemi.lorenzo89@gmail.com (L.S.); 5 Department of Anaesthesia and Intensive Care, University Hospital “G.Rodolico”, University of Catania, via Santa Sofia 78, 95123 Catania, Italy; astmar@tiscali.it (M.A.); paolomurabito@tiscali.it (P.M.)

**Keywords:** perioperative goal-directed fluid therapy, hemodynamics monitoring, fluid management, kidney transplantation, major postoperative complications, outcome of surgery

## Abstract

Background: Kidney transplantation is considered the first-choice therapy in end-stage renal disease (ESRD) patients. Despite recent improvements in terms of outcomes and graft survival in recipients, postoperative complications still concern the health-care providers involved in the management of those patients. Particularly challenging are cardiovascular complications. Perioperative goal-directed fluid-therapy (PGDT) and hemodynamic optimization are widely used in high-risk surgical patients and are associated with a significant reduction in postoperative complication rates and length of stay (LOS). The aim of this work is to compare the effects of perioperative goal-directed therapy (PGDT) with conventional fluid therapy (CFT) and to determine whether there are any differences in major postoperative complications rates and delayed graft function (DGF) outcomes. Methods: Prospective study with historical controls. Two groups, a PGDT and a CFT group, were used: The stroke volume (SV) optimization protocol was applied for the PGDT group throughout the procedure. Conventional fluid therapy with fluids titration at a central venous pressure (CVP) of 8–12 mmHg and mean arterial pressure (MAP) >80 mmHg was applied to the control group. Postoperative data collection including vital signs, weight, urinary output, serum creatinine, blood urea nitrogen, serum potassium, and assessment of volemic status and the signs and symptoms of major postoperative complications occurred at 24 h, 72 h, 7 days, and 30 days after transplantation. Results: Among the 66 patients enrolled (33 for each group) similar physical characteristics were proved. Good functional recovery was evident in 92% of the CFT group, 98% of the PGDT group, and 94% of total patients. The statistical analysis showed a difference in postoperative complications as follows: Significant reduction of cardiovascular complications and DGF episodes (*p* < 0.05), and surgical complications (*p* < 0.01). There were no significant differences in pulmonary or other complications. Conclusions: PGDT and SV optimization effectively influenced the rate of major postoperative complications, reducing the overall morbidity and thus the mortality in patients receiving kidney transplantation.

## 1. Introduction

Kidney transplantation is considered the best replacement therapy for patients with end stage renal disease (ESRD) [1]. Despite an increasing number of high-risk patients on the waiting lists, such as those suffering from older age, ischemic heart disease, diabetes, and congestive heart failure, the postoperative complication rate is relatively low compared to other solid organ transplantation procedures [1,2]. However, kidney transplant recipients experience higher postoperative morbidity and mortality rates than the general population, and cardiovascular complications represent the leading cause of death with functioning grafts, comprising up to 22% of all-cause mortality [2,3,4]. Immunosuppressive therapy, while substantially reducing the number of acute rejection episodes, leads to the increased risk of cardiovascular complications in the long-term compared with the general population [5]. The healthcare literature shows an average cardiovascular complication rate of 6%–10% in the early postoperative period. Data from a recent review carried out in the US show that the average cardiovascular complication rate in recipients of kidney transplant is quite high and is the cause of death in 17% of patients. In addition, cerebrovascular disease is the cause of death in 22% of patients [3,4]. Another review reported that 3%–17% of patients who receive a solid organ transplantation develop a major postoperative complication within one month after surgery. This is mostly acute respiratory failure, with mechanical ventilation support being required in 46.5% of cases. This is associated with 30-day and 90-day mortality rates of 22.5% [6]. Moreover, in kidney transplantation recipients, acute respiratory failure episodes are associated with an increased risk of graft loss [7]. It was recently shown that the presence of comorbidities, such as diabetes or arterial hypertension, before transplantation increases the risk of nephrological-urological postoperative complications. Similarly, the presence of an anemic condition increases the risk of cardiovascular and hematological complications in the same surgical population [8]. However, very few data are present in the literature related to the prevention of cardiovascular events during the surgical procedure and the immediate postoperative period in kidney transplant recipients by perioperative measures. Moreover, each kidney transplantation candidate often has electrolyte imbalances and tends to oscillate between hypovolemia and hypervolemia [9]. This results in a very narrow margin of safety for intravenous fluid resuscitation and maintenance, so this may increase the risk of developing delayed graft function (DGF), acute kidney injury (AKI), and fluid overload after kidney transplantation [10]. Hypovolemia can lead to further kidney injury, but excessive fluid therapy can result in pulmonary edema, so optimal fluid management is mandatory to reduce perioperative complications, particularly in patients with concomitant comorbidities such as older age, diabetes, obesity, and arterial hypertension [8,11].

During the procedure. it is crucially important to ensure a proper volemic status and simultaneous hemodynamic response of the patient in order to ensure prompt resumption of organ function at the end of transplantation. Central venous pressure (CVP)-guided volume infusion has been the traditional approach in renal transplantation [12] and involves intraoperative infusion of large volumes of fluid, on the basis of maximal volume infusion, to the point of no further fluid responsiveness [13]. However, this can lead to excess fluid infusion, which can damage the endothelial glycocalyx and lead to a fluid shift into the interstitial space [14]. For several years, during kidney transplantation, a liberal fluid-therapy attitude was recommended, with infusion rate values ranging from 10–15 mL/kg/h to 30–40 mL/kg/h with a CVP of 8–12 mmHg, in order to promote early function recovery of implanted grafts [14,15,16,17]. Over the last few years, this attitude has been downsized in favor of less aggressive fluid therapy, and infusion is now driven by relatively accurate hemodynamic indicators (CVP, mean arterial pressure [MAP]) characterized by an infusion rate of 10–15 mL/kg/h with a target CVP of 7–9 mmHg. This has resulted in a reduction in cardiovascular complications with good graft survival [18]. Moreover, it has recently been shown that it is not in itself essential to maintain a precise hemodynamic target CVP of 10–15 mmHg throughout the procedure as well as the timing of fluid challenge at a particular time of transplantation to ensure an established hemodynamic condition. All this has been shown to be associated with earlier recovery and better outcomes of the graft compared to control groups [16]. However, there is no known direct correlation between these fluid therapy regimens and the relative rates of postoperative cardiovascular, respiratory, and surgical complications. Studies on this topic are deficient and somewhat divergent, thus it is difficult to determine the potential impact of hemodynamic optimization protocols on major postoperative complication rates and organ function recovery [18,19]. In addition, an inadequate fluid therapy regimen can, in itself, potentially cause an increasing rate of postoperative complications in surgical patients without any form of renal failure [20]. Standard volemic indicators, such as static or pressure-based CVP, PCWP (pulmonary capillary wedge pressure), and MAP have been reported to show a remarkable inflection in their reliability in identifying the actual volemic status in high risk patients. Moreover, they have not been effective in identifying a fluid-responsive condition in high risk surgical patients [21,22,23]. In view of the need to maintain an adequate hemodynamic status with adequate tissue perfusion in high risk patients, it is considered appropriate to give the necessary fluids according to standardized protocols intended to maintain a predetermined hemodynamic condition. Therefore, our intent is to provide greater precision in the hemodynamic and fluid management of kidney transplantation recipients using a specific protocol, framed as an innovative concept of PGDT. In our experience, such a hemodynamic approach is achievable through implementation of the minimally invasive monitoring FloTrac^TM^/EV1000 sensor (Edwards Lifesciences LLC, Irvine, CA, USA), which has already been widely validated in high risk patients undergoing major abdominal, vascular, trauma, orthopedic, and cardiothoracic surgeries [24,25,26,27]. Primary Endpoint: To assess the effects of PGDT on the incidence rate of postoperative cardiovascular complications in kidney transplantation recipients. Secondary Endpoint: To assess the impact of PGDT on the graft loss rate, number of DGF episodes, and other postoperative complications in the same group of patients.

## 2. Materials and Methods

This prospective observational study, with historical control, included all patients who underwent kidney transplantation from January 2016 until January 2018. Ethical approval for this study (Ethical Committee Catania 1-n. 31/2016/PO) was provided by the Ethical Committee of Catania 1 “Policlinico—Vittorio Emanuele University Hospital”, Catania, Italy (Chairperson Prof. F. Drago) on 14 March 2016. All patients had to meet the following inclusion criteria to be eligible: ASA, Physical Status III-IV, age 18–65 years, first kidney transplantation, sinus rhythm, absence of atrial fibrillation (AF) or other severe arrhythmia, and completion of informed consent. Patients who underwent kidney transplantation from January 2016 to January 2018 were included in the PGDT group. The enrolled patients were not randomized as the perioperative fluid management of such patients is part of an established protocol to which all attending anesthesiologists adhere to in our institution. The PGDT group was compared with a historical cohort of patients who underwent kidney transplantation from January 2014 to December 2015 and were managed by conventional fluid-therapy (CFT group). All patients were preliminarily assessed by clinical examination, electrocardiogram, and chest X-ray. All transplantation procedures were performed by the same surgical team using a standard technique [28]. The donors’ characteristics such as age, cause of death (DBD), terminal serum creatinine level, presence of long-standing (>10 years) diabetes and/or hypertension, days spent in the intensive care unit, and cold ischemia time were evaluated. In the protocol group, hemodynamic management was implemented by the use of the FloTrac/EV1000 monitor during transplantation to adjust fluid-therapy according to a specific protocol (NICE–Kuper–SV Optimization) in association with routine monitoring. In contrast, in the control group, a conventional fluid-therapy regimen was adopted with a standardized approach for the type of procedure (CVP 8–12 mmHg, systolic blood pressure (SBP) >120/MAP >80) according to the recommendations of good clinical practice and international guidelines [29].

### 2.1. Intraoperative Phase

**PGDT Group**: All patients were monitored with electrocardiogram (ECG), non-invasive blood pressure (NIBP), SpO_2_ and bispectral index (BIS) general anesthesia was achieved by administering the following drugs in combination: 2 mg/kg propofol i.v., 2–3 mg/kg fentanyl i.v., and 0.6 mg/kg rocuronium i.v.; followed by the administration of 0.8–1.0 sevofluorane minimum aveolar concentration (MAC; BIS between 40 and 60) and 0.2/0.5 mg/kg/min remifentanil i.v. After intubation, a tidal volume of 8 mL/kg of the patients’ ideal body weight was set, with a respiratory rate of 12–14 bpm to maintain normocapnia conditions. Furthermore, a radial artery catheter was placed and connected to the FloTrac sensor, and a central venous catheter was positioned with the Seldinger technique to allow hemodynamic and CVP monitoring. Fifteen minutes after the incision by the surgeon, the first phase of the protocol to search for the optimal stroke volume index (SVI; 40–60 mL/m^2^) started. After initial annotation of the hemodynamic parameters derived from the FloTrac sensor (SVI/CI, SVV, SVRI, IBP) a crystalloid bolus of 250 mL of ringer acetate (RA) solution in 5–10 min was performed. If the SVI value had increased compared to the previous measurement of 10% or greater, a second crystalloid bolus of 250 mL of RA was performed, and the hemodynamic values were collected again. A further crystalloid bolus was performed if an SVI increase of 10% or greater occurred after the fluid challenge. We expected the maximum number of boluses to be 3. The optimal SVI (mean value of measurements performed) and trigger SVI (negative variation of >10% of optimal SVI) were defined. The second phase of the intraoperative protocol started with maintenance of the SVI above the trigger value: This was done by volume therapy with a 250 mL RA bolus only if the SVI value dropped below the SVI trigger. If the SVI value stayed above the trigger value until the end of transplantation, only a baseline rehydration therapy of 1 mL/kg/h was given. RA was infused as a fluid challenge and maintenance fluid, and NaCl 0.9% was given only for drug infusion. When severe hypotension episodes (SBP < 100 and MAP < 65 mmHg) occurred, in the presence of an adequate SVI and lower bounds of systemic vascular resistance index (SVR/SVRI; 800–1200 dynes-sec/cm^−2^ to 1970–2390 dynes-sec/cm^−2^/m), a 2 mg ethyl-ephrine bolus was given to restore normal arterial pressure values. At the end of the aforesaid procedures and after a brief observation period in the recovery room, patients were discharged from the operating block to semi-intensive monitoring area of the Transplant Unit.

**CFT Group**: All patients were monitored in a similar manner to the PGDT group. General anesthesia and hemodynamic monitoring were done in accordance with the PGDT protocol. The fluid-therapy regimen was about 10–20 mL/kg/h throughout the procedure with a potential 250/500 mL bolus used as a fluid challenge at the discretion of the attending anesthesiologist, depending on the patients’ hemodynamic status and the established targets of MAP > 80 and CVP > 8–12 mmHg. RA was infused as a fluid challenge and maintenance fluid, NaCl 0.9% was given only for drug infusion. If severe hypotension episodes (SBP < 100 and MAP < 65 mmHg) occurred, a 2 mg ethyl-ephrine bolus was given to restore normal arterial pressure values. At the end of these procedures, patients were discharged from the operating block to semi-intensive monitoring area of the Transplant Unit.

During transplantation, all patients in both groups received 100 mg of furosemide at the time of vascular declamping together with 250 mg of methylprednisone. Induction therapy with antithymocyte globulin (ATG; Fresenius, Fresenius, Bad Homburg, Germany) or anti-interleukin-2 receptor antibodies (Simulect; Novartis, Basel, Switzerland) was used in all groups for patients aged <55 years, those receiving a second transplant, or patients with >30% panel-reactive antibodies or with donor-specific antibodies (mean fluorescence intensity >3000). In the immunosuppression protocol, methylprednisolone was initiated at the time of transplantation, with a starting dose of 500 mg and then tapered to a maintenance dose of 5 mg/day by the end of a 4 month period. Mycophenolic acid was given at a dose of 1440 mg/day. For patients receiving tacrolimus-based immunosuppression, tacrolimus was initiated at 0.1 mg/kg/day, with the dose adjusted to keep the level at 10–12 ng/mL for the first month after transplantation and 8–10 ng/mL subsequently. For recipients receiving cyclosporine-based immunosuppression, cyclosporine was started the day after transplantation at 5 mg/kg/day, with the dose adjusted to keep the level at 180–200 ng/mL for the first 3 months after transplantation and 120–180 ng/mL in the next 3–6 months after transplantation. Everolimus was initiated at 1.50 mg/day beginning 5 days after transplantation with the dose adjusted to keep the level at 3–6 ng/mL.

### 2.2. Postoperative Phase

During the postoperative period, each patient was admitted in the semi-ntensive care sector of Transplant Unit and managed with a standardized fluid-therapy protocol [29]. An established monitoring procedure was performed daily unless otherwise indicated by the patients’ clinical conditions. In the event of persistent hypotension that was not linked to any bleeding condition, a norepinephrine infusion was given to target a MAP of 100 mmHg. All patients were evaluated daily by a standard monitoring procedure involving nephrological biomarkers and sonographic assessment of the graft.

The following postoperative parameters were evaluated at 24 h (T1), 72 h (T2), and 7 days (T3) after transplantation to detect of any signs and symptoms of cardiovascular (CV) complications, renal function impairment, pulmonary complications, or gastrointestinal (GI) complications: Heart rate (HR), NIBP, SpO_2_, pain numeric rating scale (NRS), patient weight, urinary output, serum creatinine, blood urea nitrogen, serum potassium, and inferior vena cava collapsibility index (IVCCI) in order to non-invasively assess the patients’ volemic status. Patients were studied and analyzed for up to seven days after transplantation in order to identify any major CV complications (acute coronary syndromes, congestive cardiac failure, stroke or transitory ischemic attack), renal complications (DGF and acute rejection), pulmonary complications (acute pulmonary edema, acute respiratory distress), or GI complications (postoperative ileus and postoperative nausea and vomiting). The CV complications identified during the evaluation were categorized as follows: Acute coronary syndromes (ACS) with ST-segment elevation and increased troponin levels; ACS without ST segment elevation associated with atrial fibrillation; and congestive cardiac failure. We considered ACS to include any sign or symptom associated with chest pain or discomfort, including pressure, tightness or fullness, and pain or discomfort in one or both arms, the jaw, neck, back or stomach. Confirmation by an electrocardiogram (ECG) to measure the heart’s electrical and blood sampling of myocardial damage biomarkers were used as diagnostic test for acute coronary syndrome [30]. DGF was defined as the need to undergo a hemodialysis session within the first week after transplantation. After 30 days post-transplantation, an additional morbidity evaluation to detect other or residual postoperative complication’s episodes was carried out.

### 2.3. Statistical Analysis

Given the observational nature of study, which included a historical comparison, and based on estimation of average effect size of 0.30 with an alpha level of 0.05 and a statistical power (1-beta) of 0.80 regarding the incidence rate of major postoperative complications, we estimated that at the least 88 patients were required, with 44 patients enrolled in the PGDT group and another 44 in the CFT group. After two years of data collection and through an ad interim analysis performed on 66 patients, 33 in the PGDT group, and 33 in the CFT group, a new average effect size of 0.50 was estimated with an alpha of 0.05 and a statistical power (1-beta) of >0.90. Thus, having already reached an appropriate number of patients to validate our assumptions, we carried out a comprehensive statistical analysis.

A descriptive statistical analysis was conducted for qualitative data using the absolute and percentage frequencies of the above-mentioned complications in each group. For normally distributed quantitative data, the means and standard deviations were used. For non-normally distributed quantitative data, the medians and the interquartile ranges (IQR) were used.

For comparisons between the two groups, we used the chi-square test with Yates correction or the Fisher’s exact test.

## 3. Results

The patients’ general characteristics (33 in the PGDT group and 33 in the CFT group) were similar between groups (Table 1). The age, sex distribution, and body mass index (BMI) were similar between the two groups while time spent on dialysis and on waiting lists was slight lower in the PGDT group without reaching statistical significance. Polycystic kidney disease was the leading cause of ESRD in both groups, but there was a higher prevalence of diabetes and diabetic nephropathy in the PGDT group (*p* < 0.05). Arterial hypertension was present in 90% of PGDT patients and in 84.8% of controls (*p* = NS). The donor characteristics were similar between groups, although PGDT donors had a significantly higher prevalence of diabetes. However, the function of donor kidneys was excellent in both groups with a terminal serum creatinine level of 0.8 ± 0.23 in the PGDT group and 0.73 ± 0.19 in the control group (*p* = NS). The mean time taken for transplantation was 155 ± 24 min in the CFT group and 148 ± 25 min in the PGDT group. Throughout the procedure, the total amount of fluid given was 1000 (250) mL in the CFT group vs. 980 (700) mL in the PGDT group. Curiously, despite a similar total fluid amount being infused in the two groups, substantial intraperson variability was observed in the PGDT group as confirmed by available statistical dispersion values (IQR: 700 vs. 250; MAD: 414.4 vs. 186.4), in accordance with the “tailored” approach derived from PGDT implementation (Table 2 and Figure S1A–C). The main intraoperative characteristics of both groups are shown in Table 2.

**Figure 1:**
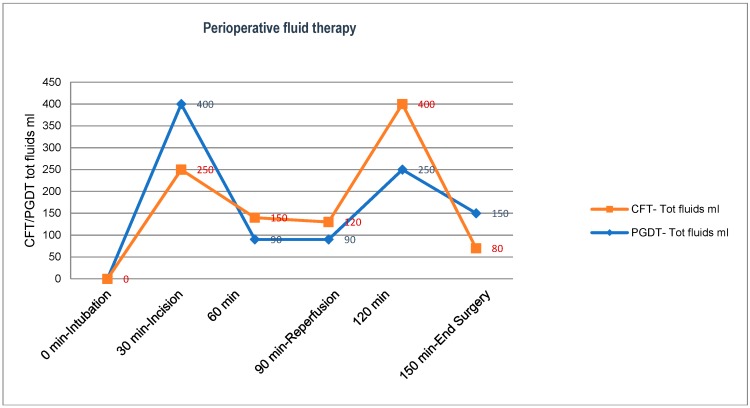
Comparison of perioperative fluid management between the conventional fluid therapy (CFT) vs. perioperative goal-directed fluid therapy (PGDT) group. CFT—Tot fluids: total perioperative fluid therapy in the CFT group; PGDT—Tot fluids: total perioperative fluid therapy in the PGDT group; min: minutes of surgery.

Main preoperative data:

Intraoperative data:

The number of fluid challenges performed during the pre-incisional phase or after the declamping of vascular anastomosis in the reperfusion phase was on average 2.1 (Figure 1). There were no cases of severe hypotension (MAP < 65 mmHg) during the procedure in either groups; moderate hypotension episodes were reported (65–80 mmHg) in 11 cases (33.3%) in the PGDT group and 12 cases (34.5%) in the CFT group. These were mostly unique and occurred after reperfusion of the graft; they were treated effectively with a single bolus of ethyl-eprhine. The total urinary output at the end of surgery was 100 (650) mL in the PGDT group and 100 (400) mL in the CFT group. The mean postoperative data are shown in Table 3.

Moreover, during the postoperative period, a volemic assessment, the Caval Collapsibility Index, was performed, and the prevalence of patients analyzed at 24 h, 72 h and 7 days after surgery was judged to be hemodynamically adequate (IVCCI < 50%), with a mean IVCCI value of 32% in spontaneous breathing and without any contraindications to that evaluation (severe dyspnea, right ventricular hypertension, or pericardial effusion). A significant difference in the incidence rate of postoperative CV complications was shown, in regard to the overall number of ACS events in the PGDT and CFT groups in the first postoperative week, with 1 (3%) vs. 6 (18%) (*p* < 0.05), respectively. More precisely, in dealing with the reported ACS episodes in the PGDT group, we identified *n* = 1 ACS without ST-segment elevation associated with atrial fibrillation (3%), whereas in the CFT group, there were 2 ACS with ST-segment elevation and increased troponin levels (8%) and 4 ACS cases without ST-segment elevation associated with atrial fibrillation (12%), which is in line with recently reported data [31]. The 30-day ACS incidence rate was lower in the PGDT than in the CFT group (*n* = 0 vs. 2 (6%) *p* = NS). Other cardiovascular complications did not show significant differences in incidence rate between the PGDT and CFT groups (Table 4). The incidence of postoperative pulmonary complications was very low, with only one case (3%) in both groups (*p* = NS; Table 5). PGDT patients showed a lower incidence of DGF with 4 (12.1%) vs. 11 (32.5%) cases in the CFT group (*p* < 0.05), and the duration of DGF was lower in the PGDT group (Table 6). The reduction in DGF incidence in the PGDT group resulted in significantly better 7-day (2.2 mg/dL vs 2.8 mg/dL, *p* < 0.05) and 30–day (1.35 mg/dL vs. 1.55 mg/dL, *p* < 0.05) mean serum creatinine levels compared to the control group (Table 1, Table 2 and Table 3). Consistent data emerged from the analysis of postoperative ileus prevalence between the two groups at 24 h and 72 h after transplantation, with a lower rate in the PGDT group compared to the CFT group (Table 6). A small but not significant difference in postoperative nausea and vomiting (PONV) incidence was observed between the two groups (*p* = NS). The 30-day morbidity data, including all postoperative residual or new onset complications, are shown in Table 7.

## 4. Discussion

Adequate perfusion of the transplanted kidney is required to avoid hypoxia, the leading cause of organ dysfunction. The fundamental role of health care professionals involved in perioperative fluid management of kidney transplantation is to identify the perfect balance of fluid therapy. Accumulating evidence supports the concept that fluid therapy should be individualized and based on dynamic indices of the intravascular volume [10]. Dynamic variation in the arterial waveform-derived parameters systolic pressure variation (SPV), pulse pressure variation (PPV), and stroke volume variation (SVV) in mechanically ventilated patients are currently the most precise predictors of fluid responsiveness, particularly when compared to static parameters [10,32].This study revealed how the “tailored” approach, achieved through the implementation of PGDT strategies during kidney transplantation, might considerably decrease the incidence rate of major perioperative complications and increase the survival rate of the graft. Today, it is widely shared and supported by a large number of publications, as such hemodynamic monitoring systems could represent a key resource to reduce major postoperative complications and therefore short and long term morbidity and mortality [33,34,35,36,37]. In the PGDT group, postoperative CV complications, namely ACS, new acute. or chronic re-acutization of cardiac failure episodes, substantially reduced. Indeed, this turns out to be a very good result for high cardiovascular risk patients, whose cardiovascular disease remains the leading cause of death with a functioning graft and therefore is a leading cause of graft failure [37]. This is likely also related to the lower incidence of DGF in the PGDT group, suggesting that immediate graft function is the key to reducing the incidence of early postoperative complications, particularly in high-risk recipients. Although this study did not investigate the effect of PGDT on long-term complications, the role of DGF in terms of the reduction of long-term grafts and patient survival is well documented [28,38,39,40].

There was also a decreased incidence rate of DGF episodes during the first postoperative week. The determinism of phenomena such as DGF is notoriously complicated due to several factors that are partly related to the pathophysiological characteristics of the recipient and the compensation of comorbidities, even in the preoperative phase. It is known that the occurrence of DGF episodes has a detrimental effect on graft survival _(11)_. In this trial, the incidence of DGF was statistically lower in the PGDT group than in controls, suggesting that correct fluid management during kidney transplantation may reduce the rate of ischemia/reperfusion injury and, therefore, increase the likelihood of immediate graft function. This would probably be reflected in better long-term outcomes given that DGF is a strong risk factor for early recipient death and graft loss during the first year post-transplant [41].

As far as major surgical complications are concerned, the difference in incidence rates between the two groups further highlighted the remarkable role of the PGDT protocol. The adequate hemodynamic status obtained with the PGDT protocol allowed for a faster recovery from surgery, as demonstrated by the lower incidence of postoperative ileus at 24 h and 72 h after surgery in the PGDT compared to the control group.

Although this pilot study provides a step forward in the knowledge and management of fluid therapy in kidney transplantation, we are conscious of its limitations. For example, the study sample was relatively small and not randomized but included a single series of kidney transplants performed at a single institution, thus eliminating potential confounding factors such as race, different surgical procedures, and different postoperative protocols. The PGDT protocol was applied only during the intraoperative phase, demanding the preoperative and postoperative fluid and hemodynamic management be completed by the surgical team of the Transplant Unit. However, the postoperative management followed a standardized protocol, and there were no differences in postoperative fluid management between cases enrolled both in the PGDT group compared to the CFTgroup, suggesting that the significant reduction in the incidence of DGF and cardiovascular complications could have been largely influenced by the PGDT approach. Certainly the identification of postoperative fluid-responders (IVCCI > 50%) may stimulate the use of this approach even in the postoperative period.

## 5. Conclusions

Although the effective perioperative fluid administration in kidney transplantation remains challenging, this pioneering trial showed promising evidence that the use of individualized approach adapted to each patient’s perioperative needs and responses to volume therapy might help to reduce the incidence of delayed graft function and, consequently, the incidence of cardiovascular and general complications in kidney transplant recipients.

Indeed, these results call for further trials on a more extensive group of kidney transplantation recipients to confirm our exploratory data, perhaps in randomized prospective single or multicenter studies. Further trials with larger cohorts of patients should also investigate if this “tailored” approach may be useful for improving the long-term outcomes of kidney transplantation.

## Figures and Tables

**Table 1:** PGDT: perioperative goal directed therapy; CFT: conventional fluid therapy; BMI: body mass index; ESRD: end-stage renal disease; TAC: tacrolimus; MPA: mycophenolic acid; Ster: steroids; CyA: cyclosporine; EVE: everolimus; DGF: delayed graft function.

Characteristics	PGDT	CFT	*p*-Value
N	33	33	
Age (years)	50 ± 9.7	53 ± 10	0.638
Sex (M/F)	20/13	19/14	0.732
BMI (kg/m^2^)	24.8 ± 5	24.3 ± 4.2	0.443
Waiting list (months)	23.9 ± 18.1	27.1 ± 21.4	0.434
**Recipient cause of ESRD (*n*)**			
Polycystic kidney disease	10 (33.3%)	9 (27.3%)	0.543
Diabetes	8 (24.3%)	3 (9%)	**<0.05**
Other/unknown	15 (45.4%)	21 (63.6%)	**<0.05**
Pre-transplant dialysis (months)	40.4 ± 32.1	46.7 ± 32	0.328
Hemodialysis/peritoneal dialysis	26/7	30/2	0.184
**Recipient comorbidities (*n*)**			
Hypertension	30 (90%)	28 (84.8%)	0.543
Previous acute myocardial infarction	5 (15.1%)	4 (12.1%)	0.388
Donor age (yr)	51.7 ± 16	52.1 ± 15.7	0.264
**Donor Comorbidities (*n*)**			
Hypertension	12 (36.3%)	11 (33.3%)	0.953
Diabetes	7 (21.2%)	1 (3%)	**<0.05**
Donor intensive care unit stay	5.2 ± 2.1	5.2 ± 3.5	0.825
Terminal serum creatinine (mg/dL)	0.8 ± 0.2	0.7 ± 0.2	0.896
Cold ischemia time (min)	822 ± 370.2	744 ± 376.1	0.243
Operative time (min)	148.2 ± 71.7	157.9 ± 46.1	0.723
**Immunosuppression (*n*)**			
Induction	18 (54.5%)	17 (51.5%)	0.742
Tac + MPA + Ster	25 (75.7%)	24 (72.7%)	0.456
CyA + MPA + Ster	3 (9%)	2 (6%)	0.765
Ever + Tac + Ster	5 (15.1%)	7 (21.2%)	0.523
Blood loss (mL)	300 ± 122	322 ± 142	0.221
DGF	4 (12.1%)	11 (32.5%)	**<0.05**
Duration of DGF (days)	3.2 ± 1.2	8.3 ± 3.5	**<0.05**
Acute rejection	1 (3%)	1 (3%)	1
Postoperative hospital stay (days)	14.5 ± 6.1	13.6 ± 16.5	0.321
Mean 7-day serum creatinine (mg/dL)	2.23	2.85	**<0.05**
Mean 30-day serum creatinine (mg/dL)	1.35	1.55	**<0.05**

**Table 2:** SBP/DBP: Systolic/diastolic blood pressure; MAP: mean arterial pressure; SVI: stroke volume index; CI: cardiac index; IQR: interquartile range; RA: ringer acetate; NaCl 0.9%: normal saline.

Intraoperative	PGDT Group	CFT Group	*p* Value
SBP/DBP	114 ± 10/61 ± 7 mmHg	109 ± 8/61 ± 5 mmHg	0.3/0.2
MAP	78 ± 2 mmHg	77 ± 2 mmHg	0.2
SVI	51 ± 4 mL/min/m^2^	/	/
CI	4.2 L/min/m^2^	/	/
Tot fluids (median-IQR)	980 (700) mL	1000 (250) mL	0.2
RA/NaCl 0.9%	830 vs. 150 mL (85% vs. 15%)	800 vs. 200 mL (80% vs. 20%)	/
Urine output (median-IQR)	100 (650) mL	100 (400) mL	0.09

**Table 3:** Postoperative data. T1: 24 h; T2: 48 h; and T3: 7 days after transplantation.

	CFT Group			PGDT Group			*p* Value
Average Values	T1	T2	T3	T1	T2	T3	T1/T2/T3
**Serum Creatinine, mg/dL**	5.5 ± 2.5	5.4 ± 2.4	2.8 ± 1.4	7.7 ± 3.1	6.4 ± 3.1	2.2 ± 1.5	**<0.05**/0.08/**<0.05**
**Blood Urea Nitrogen, mg/dL**	143 ± 47	143 ± 47	132 ± 47	125 ± 50	140 ± 50	140 ± 54	0.06/0.4/0.3
**Serum potassium, mmol/L**	4.3 ± 0.5	4.3 ± 0.6	4.1 ± 0.3	4.5 ± 0.8	4.1 ± 0.7	4.2 ± 0.6	0.07/0.1/0.4
**SpO_2_ %**	95 ± 2	96 ± 1.8	97 ± 1.5	95 ± 2	95 ± 2.1	98 ± 1.8	0.5/0.09/0.07
**NIBP**	131 ± 17/74 ± 10	129 ± 14/74 ± 12	131 ± 14/73 ± 9	134 ± 19/75 ± 12	135 ± 16/75 ± 9	135 ± 17/77 ± 10	0.2–0.3/0.06–0.3/0.2–0.06
**Heart rate, bpm**	83 ± 8.2	82 ± 9.6	80 ± 7.1	81 ± 11	82 ± 11	77 ± 10	0.1/0.4/0.06
**Urinary output, mL/h**	131 ± 66	131 ± 65	95 ± 47	125 ± 120	119 ± 68	97 ± 46	0.1/0.06/0.2

**Table 4:** CV: Cardiovascular; ACS: acute coronary syndromes; PGDT: protocol group; CFT: control group; complications+: evidence of complications; complications−: no complications; T3: first week.

CV Complications *ACS*	Complications+ (T3)	Complications− (T3)	CV Complications− *Congestive Failure*	Complications+ (T3)	Complications− (T3)
PGDT (*n*)	1 (3%)	32 (97%)	PGDT (*n*)	0 (0%)	33 (100%)
CFT (*n*)	6 (18.2%)	27 (81.8%)	CFT (*n*)	1 (3%)	32 (97%)
Total (*n*)	7 (9%)	59 (91%)	Total (*n*)	1 (1.5%)	65 (98.5%)
Chi-square Fisher’s exact test	*p* < 0.05	/	Chi-square Fisher’s exact test	*p* > 0.05	/

**Table 5:** RDS Respiratory distress syndromes; PGDT: protocol group; CFT: control group; complications+: evidence of complications; complications−: no complications; T3: first week.

Pulmonary Complications *RDS*	Complications+ (T3)	Complications− (T3)	Pulmonary Complications Pneumonia	Complications+ (T3)	Complication− (T3)
PGDT (*n*)	0 (0%)	33 (100%)	PGDT (*n*)	1 (3%)	32 (97%)
CFT (*n*)	1 (3%)	32 (97%)	CFT (*n*)	0 (0%)	33 (100%)
Total (*n*)	1 (1.5%)	65 (98.5%)	Total (*n*)	1 (1.5%)	65 (98.5%)
Chi-square Fisher’s exact test	*p* > 0.05	/	Chi-square Fisher’s exact test	*p* > 0.05	/

**Table 6:** DGF: delayed graft function; GI: gastrointestinal; PGDT: protocol group; CFT: control group; complications+: evidence of complications; complications−: no complications. T3: first week; T2: 72 h.

*DGF*/*Hemodialysis*	Complications+ (T3)	Complication− (T3)	GI Complications *Postoperative Ileus*	Complications+ (T2)	Complications (T2)
PGDT (*n*)	4 (12.1%)	29 (87.9%)	PGDT (*n*)	3 (9.1%)	30 (90.9%)
CFT (*n*)	11 (33%)	22 (67%)	CFT (*n*)	26 (78.8%)	7 (21.2%)
Total (*n*)	15 (23%)	51 (77%)	Total (*n*)	29 (43.9%)	37 (56.1%)
Chi-square Fisher’s exact test	*p* < 0.05	/	Chi-square Fisher’s exact test	*p* < 0.01	/

**Table 7:** PGDT: protocol group; CFT: control group.

30-Day Morbidity	Cardiovascular Complications	Renal Complications	Pulmonary Complications
PGDT	0 (0%)	1 (3%)	0 (0%)
CFT	2 (6%)	1 (3%)	1 (3%)
Total	2 (6%)	2 (6%)	1 (3%)
/	*p* > 0.05	*p* > 0.05	*p* > 0.05

## Supplementary Materials

The following are available online at http://www.mdpi.com/2077-0383/8/1/80/s1, Figure S1: Fluid therapy amount distribution.

## Abbreviations

ACS	acute coronary syndromes
AF	atrial fibrillation
AKI	acute kidney injury
ASA	American Society of Anesthesiology
ATG	anti thymocyte globulin
BIS	bispectral index
BMI	body mass index
BUN	blood urea nitrogen
CFT.	conventional fluid therapy
CI	cardiac index
CIT	cold ischemia time
CyA	cyclosporine
CV	cardiovascular
CVP	central venous pressure
DBP	diastolic blood pressure
DGF.	delayed graft function
ECG.	electrocardiogram
ESRD.	end stage renal disease
EtCO_2_	end tidal CO_2_
EVE	everolimus
GI	gastrointestinal
HR	heart rate
ICU.	intensive care unit
IBP	invasive blood pressure
IQR	interquartile range
IVCCI	inferior vena cava collapsibility index
LOS	length of stay
MAC	minimum alveolar concentration
MAP	mean arterial pressure
MPA	mycophenolic acid
NaCl	0.9% normal saline 0.9%
NIBP	non-invasive blood pressure
NICE	National Institute of Health and Care Excellence
NRS	numeric rating scale
NS	not significative
PCWP	pulmonary capillary wedge pressure
PGDT.	perioperative goal directed therapy
PONV	postoperative nausea and vomiting
PPV	pulse pressure variation
RA	ringer acetate
RR	respiratory rate
SBP	systolic blood pressure
SpO_2_	peripheral saturation of oxygen
Ster	steroids
SV	stroke volume
SVI	stroke volume index
SPV	systolic pressure variation
SVV	stroke volume variation
SVR/SVRI	systemic vascular resistance index
TAC	tacrolimus
v	train of four
