# Supplementary figures and images for: Perioperative Goal-Directed Therapy during Kidney Transplantation: An Impact Evaluation on the Major Postoperative Complications
Source: J Clin Med. 2019 Jan 11;8(1):80. doi: 10.3390/jcm8010080 (PMC6351933; doi:10.3390/jcm8010080)

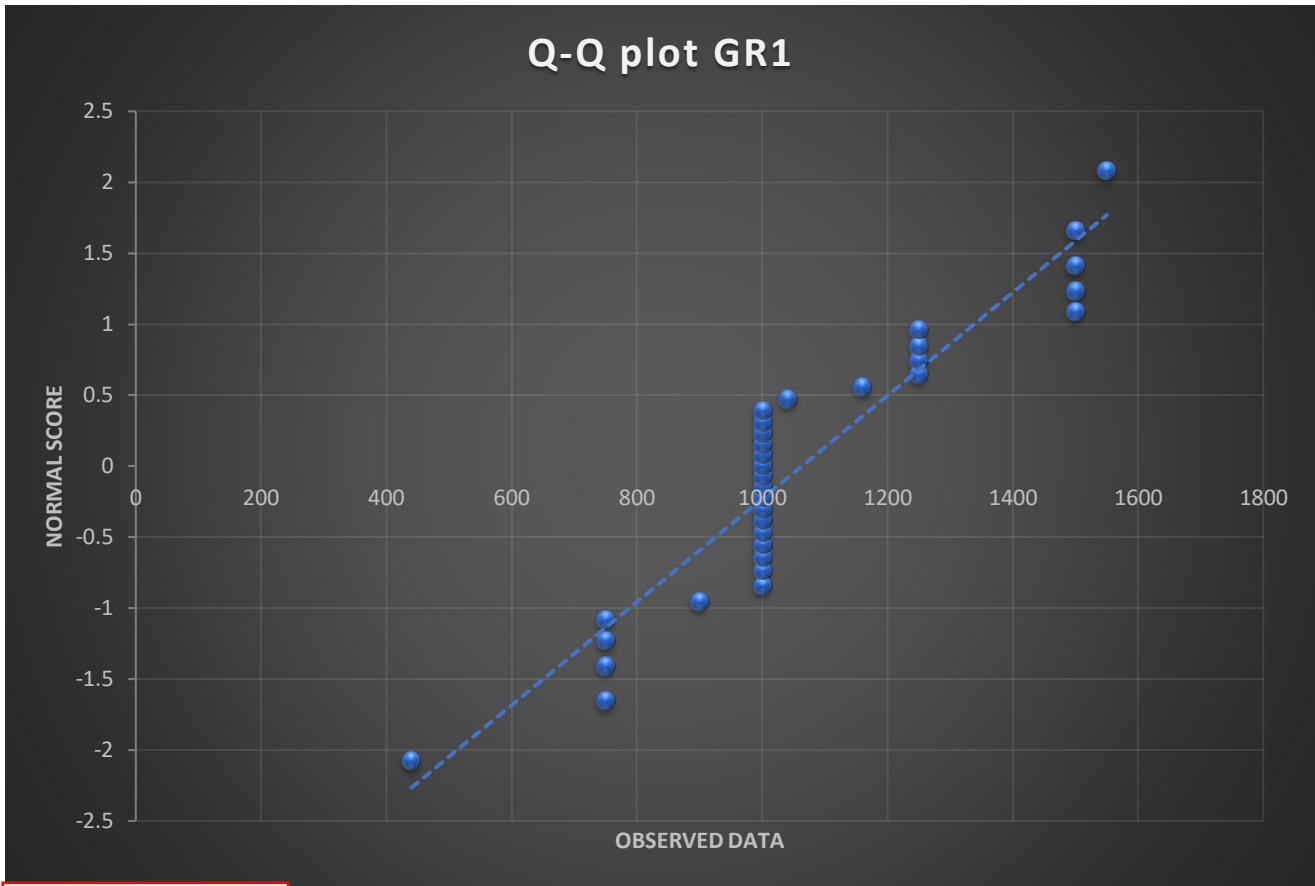

Figure 1-A. GR 1= CFT;

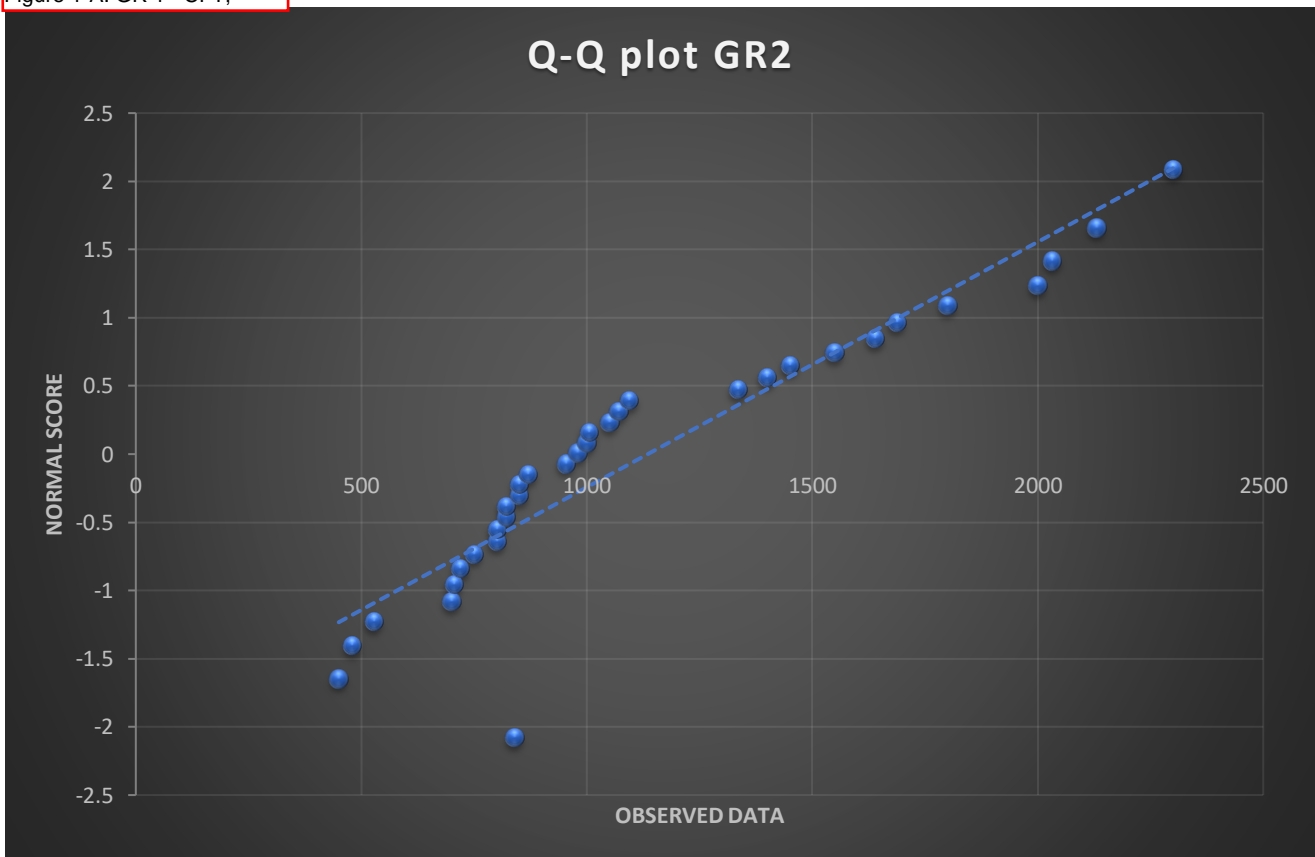

Figure 1 - B. GR 2= PGDT;

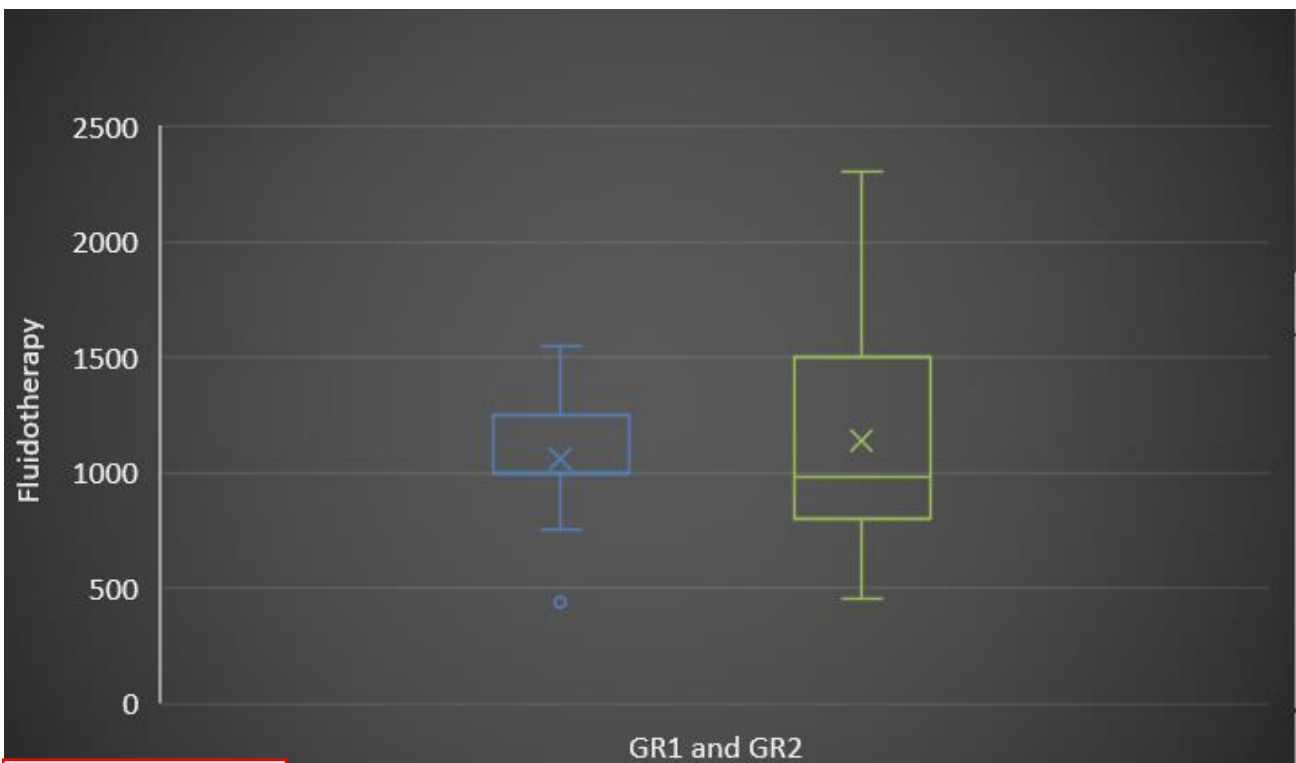

Figure 1 - C. GR 1= CFT;  
GR 2= PGDT;

Supplement: Supplementary file 1 [file jcm-08-00080-s001.pdf]
